# *Xenopus* embryos show a compensatory response following perturbation of the Notch signaling pathway

**DOI:** 10.1016/j.ydbio.2019.12.016

**Published:** 2019-12-30

**Authors:** Grace E. Solini, Mark E. Pownall, Molly J. Hillenbrand, Claire E. Tocheny, Sudip Paudel, Andrew D. Halleran, Catherine H. Bianchi, Ryan W. Huyck, Margaret S. Saha

**Affiliations:** 1 Department of Biology, College of William and Mary, Williamsburg, VA, 23185, USA; 2 These authors contributed equally.; 3 Current Address: Department of Genetics, Yale University School of Medicine, New Haven, CT 06510.; 4 Current Address: University of Cincinnati College of Medicine, Cincinnati, OH 45267.; 5 Current Address: University of Massachusetts Medical School, Worcester MA 01655.; 6 Current Address: Department of Bioengineering, California Institute of Technology, Pasadena CA 91125.; 7 Current Address: College of Education, Auburn University 36849; 8 Current Address: Eastern Virginia Medical School, Norfolk, VA 23507.

**Keywords:** Compensation, Robustness, Plasticity, Xenopus, Notch, Neural

## Abstract

As an essential feature of development, robustness ensures that embryos attain a consistent phenotype despite genetic and environmental variation. The growing number of examples demonstrating that embryos can mount a compensatory response to germline mutations in key developmental genes has heightened interest in the phenomenon of embryonic robustness. While considerable progress has been made in elucidating genetic compensation in response to germline mutations, the diversity, mechanisms, and limitations of embryonic robustness remain unclear. In this work, we have examined whether *Xenopus laevis* embryos are able to compensate for perturbations of the Notch signaling pathway induced by RNA injection constructs that either upregulate or inhibit this signaling pathway. Consistent with earlier studies, we found that at neurula stages, hyperactivation of the Notch pathway inhibited neural differentiation while inhibition of Notch signaling increases premature differentiation as assayed by neural beta tubulin expression. However, surprisingly, by hatching stages, embryos begin to compensate for these perturbations, and by swimming tadpole stages most embryos exhibited normal neuronal gene expression. Using cell proliferation and TUNEL assays, we show that the compensatory response is, in part, mediated by modulating levels of cell proliferation and apoptosis. This work provides an additional model for addressing the mechanisms of embryonic robustness and of genetic compensation.

## 1. Introduction

While all organisms possess some ability to respond and adapt to changing genetic and environmental conditions throughout their life-span, embryonic development is characterized by a particularly remarkable degree of plasticity (Bateson et al., 2014; Gilbert et al., 2015; Schwab et al., 2019). Despite phylogenetic differences, embryos typically display an exceptional robustness allowing them to respond to the considerable range of perturbations they continually face during development yet still achieve a reasonably consistent phenotype. However, while in many instances the embryo will respond with a strong compensatory response ameliorating the effects of the perturbation, in other situations, the effects of the perturbation will remain, or even become amplified throughout development (Schlosser et al., 2008; Yan et al., 2009b; Vandenberg et al., 2012; Riddiford and Schlosser, 2017; Ochi et al., 2017). Thus an embryo’s ability to generate a compensatory response has clear limitations. The nature of the response will, expectedly, also depend upon the type of perturbation as well as whether the perturbation is chronic, such as a germline genetic mutation or a continual exposure to a teratogen, or an acute perturbation such as limited exposure to a toxic compound or microinjection of a construct that disappears or becomes progressively diluted over time.

Recently there has been a growing interest in understanding the nature of genetic robustness and the mechanisms underlying the associated compensatory responses, spurred in part by the observation that phenotypes from gene knockdowns frequently display more severe phenotypes than those associated with mutations in the same gene (El-Brolosy and Stainier, 2017; Ma et al., 2019; Dooley et al., 2019; El-Brolosy et al., 2019; Young et al., 2019). This body of work has uncovered novel mechanisms including the role of mRNA degradation, gene regulatory network activation of paralogous genes, and growth compensation, among others.

Despite significant progress and growing interest in addressing this longstanding issue, our understanding of the robustness and the associated processes that allow embryos to generate compensatory responses to perturbations still remains limited (Bateson et al., 2014; Sultan, 2017; Wu and Belmonte, 2016; Yang et al., 2018; El-Brolosy and Stainier, 2017). Given that many of these recent studies have focused on germline mutations in genetically tractable organisms, investigation of additional models and organisms will help to expand our knowledge of the constraints and diversity of robustness. For example, in addition to gene knockdowns and mutations, injection of mRNA overexpression or antimorphic constructs are well-established forms of perturbation that can serve as useful models for understanding robustness (Vize et al., 1991).

While many noteworthy studies have perturbed embryonic development by microinjection of gain- and loss-of-function constructs, most have focused on the relatively immediate effects, specifically the point at which developmental aberrations appear, with the goal of assessing the function of given genes. Relatively few of the mRNA injection experiments have specifically examined how embryos respond over more extended periods of time and assayed for the presence and degree of robustness, that is, whether the embryo responds with amplifying, retaining, or compensating for the initial perturbation.

Here, in an effort to develop an additional model system for investigating the phenomenon of embryonic robustness, we asked whether *Xenopus* embryos display a compensatory response in the developing nervous system following perturbation of the Notch signaling pathway, a highly conserved juxtacrine pathway involved in a wide array of developmental processes, including early neural development (Chitnis et al., 1995; Wettstein et al., 1997). We hyperactivated the Notch signaling pathway with mRNA injections of the intracellular domain (ICD) of Notch, the region of the protein that once bound to Suppressor of Hairless translocates to the nucleus to activate new transcription of downstream effector genes. Notch signaling was downregulated with mRNA injections of a DNA-binding mutant (DBM) of Suppressor of Hairless which, when overexpressed, sequesters ICD and effectively inhibits downstream Notch signaling (Chitnis et al., 1995; Wettstein et al., 1997). Using *tubb2b, pcna*, and TUNEL to characterize the response, we show that embryos, over time, exhibit a robust compensatory response at the genetic level to both hyperactivation and downregulation of Notch signaling, one that employs both differential proliferation as well as apoptosis of cells in neural regions as likely mechanisms for mediating this compensatory response.

## 2. Materials and methods

### 2.1. Animal use

All animal care and use procedures were performed in accordance with the regulations set forth by the College of William & Mary Institutional Animal Care and Use Committee (IACUC). Embryos were obtained by natural mating of adult *X. laevis* as previously described (Sive et al., 2007) and were staged according to Nieuwkoop and Faber (1994).

### 2.2. RNA microinjections

Capped sense RNA was synthesized using the mMessage mMachine SP6 transcription kit (Ambion) and purified using the RNeasy MinElute Cleanup kit (Qiagen) according to the manufacturer’s protocol. Injections were performed in several different ways. For unilateral injections, a single blastomere was injected at the two-cell stage and embryos were reared to desired stages (mid-neurula, tailbud, or swimming tadpole). We performed unilateral injections so that the contralateral side could serve as an internal negative control, an aspect of *Xenopus* that is widely featured in the literature. While Danilchik and Black (1987) reported that the plane of first cleavage has a random orientation with respect to the future dorsal midline (Danilchik and Black, 1987), it is generally accepted that the first cleavage plane does closely correspond to the future midsaggital axis of the embryo (Klein, 1987; Masho, 1990; Blum and Ott, 2018; Kha et al., 2018; Lasser et al., 2019; Nenni et al., 2019). However to ensure that the embryos we injected unilaterally into one blastomere uniformly labeled only one side of the future embryo, embryos were examined for tracer expression at late gastrula/early neurula stages, and then again at late neurula, tailbud, or swimming tadpole stages. In 98% of the cases (n = 1194 embryos from 10 clutches, that is, different matings), expression was restricted to a single side of the embryo. Any embryo that did not label only one half of the embryo was not included in our analysis since this would obviously bias the interpretation of the results. As an additional control, the uninjected side was also compared to wild type embryos.

For unilateral injections, 4.6 nl of nuclease-free water (NFW) containing 1.5 ng or 0.75 ng of capped transcript encoding either ICD (Chitnis et al., 1995) or DBM (Wettstein et al., 1997) and 0.5 ng of GFP (Chalfie et al., 1994) or *ß-Galactosidase* (Chitnis et al., 1995) as an injection tracer was injected into one side of the embryo. All constructs were linearized with NotI and transcribed with SP6 RNA polymerase. Vehicle-injected controls were injected with 4.6 nl NFW containing 0.5 ng of GFP or ß-Galactosidase. For dosage experiments embryos were unilaterally injected with 3.0 ng of ICD or DBM in 9.2 nl NFW. Bilaterally injected embryos were injected with 4.6 nl NFW containing either 0.75 ng or 1.5 ng ICD or DBM mRNA and 0.5 ng GFP mRNA into each cell at the two-cell stage. Control embryos were injected with 1.5 ng of GFP mRNA on each side to ensure that the GFP mRNA had no effects on phenotype. ICD and DBM constructs were the kind gifts of C. Kintner.

### 2.3. Whole-mount in situ hybridization

Antisense RNA probes labeled with either digoxigenin-11 rUTP or fluorescein-12 rUTP (Roche) were synthesized as described by Sive et al. (2000) for the following genes: *tubb2b* (linearized with BamHI, transcribed with T7) (Klein et al., 2002), *sox2* (linearized with EcoRV, transcribed with T7) (Huyck et al., 2015), and *pcna* (linearized with XhoI, transcribed with T7) (Huyck et al., 2015). PCNA *in situ* hybridization (ISH) was selected based on reports in the literature suggesting that it represents an effective and accurate tool for assessing dynamic changes in proliferation (Muskhelishvili et al., 2003; Kohler et al., 2005). Whole-mount chromogenic *in situ* hybridization (ISH) using nitrobluetetrazolium/5-bromo-4-chloro-3-indolyl phosphate (NBT/ BCIP) alkaline phosphatase substrates was performed as described by Sive et al. with minor modifications (Sive et al., 2000). Embryos were photographed using an Olympus SZH10 microscope and an Olympus DP71 camera or Nikon SMZ800N microscope and Nikon DSi-R2 camera. Global image adjustments were made using Adobe Photoshop CC to correct brightness, contrast, and color balance.

### 2.4. TUNEL assay

Whole-mount terminal deoxynucleotidyl transferase (TdT) dUTP nick-end labeling (TUNEL) was carried out as described (Hensey and Gautier, 1998) with minor modifications. Following fixation in MEMFA (100 mM MOPS pH 7.4, 2 mM EGTA, 1 mM MgSO_4_, 3.7% (v/v) formaldehyde), embryos were rehydrated and permeabilized in PBS with 0.1% Tween-20, equilibrated in TdT buffer (Sigma) at room temperature (RT), and then incubated overnight in TdT buffer with digoxigenin-11 dUTP and TdT enzyme (Sigma). The reaction was stopped by incubating in 100 mM EDTA in PBS at 65 °C. An alkaline-phosphatase conjugated anti-dig antibody (Roche) was used with NBT/BCIP color reaction as in ISH.

### 2.5. Histology

Following whole-mount assays, embryos were paraffin or cryosectioned as previously described (Pownall and Saha, 2018). Results of chromogenic assays were photographed using an Olympus BX60 scope attached to a Media Cybernetics QCapture digital camera. Focusing specifically on cells within the developing central nervous system, our analysis consisted of counting the number of cells in neural tissue, as well as quantifying the number and percentage of both apoptotic and proliferative cells within the neural tube. For every experiment every section of each embryo (typically 3–4 slides) was analyzed as follows. Neural cells were quantified by identifying the boundary of the neural tube followed by counting DAPI-stained nuclei within the boundaries of neural tissue for every section. Nuclei counting was automated using a custom ImageJ script (Schneider et al., 2012) (courtesy of Samuel Clamons) based on the ITCN plug-in (Centre for Bio-Image Informatics, University of California). Data were normalized to account for variation in embryo size and sectioning by taking the total number of neural cells for each embryo and dividing by the number of sections for that embryo. This resulted in an average number of cells per section for the injected and uninjected sides, which represents a single data point on the plots in Fig. 4A and B. For the analysis of apoptotic (using the TUNEL assay) and proliferative (using PCNA ISH) cells, positive cells were identified by comparison with the images on the negative control slides; cells were designated as positive if two double blind evaluators unambiguously identified the cells as significantly above the background levels of the negative control slides. All sections were analyzed for each embryo. To normalize for any variation in embryos, the total number of PCNA or TUNEL-positive cells for the injected or uninjected side of a single embryo was divided by the total number of cells in the manually defined neural region of the corresponding injected or uninjected side of that embryo. This resulted in the fraction of PCNA or TUNEL-positive cells for that embryo; this represents a single data point in the plots in Fig. 4 C–F. Each data point represents one side of a single embryo. Statistical significance was determined with a two-tailed paired *t*-test to compare the normalized levels of positive cells in injected and uninjected sides using GraphPad Prism.

### 2.6. Morphological analysis

We also performed a qualitative morphological assay on the embryos at tailbud and swimming tadpole stages grouping embryos into normal or nearly normal (normal touch responsiveness and swimming behavior) and malformed/unresponsive (abnormal swimming behavior, significant abnormalities). A two-tailed Fisher’s Exact test in GraphPad was used to determine statistical significance.

### 2.7. Real time qRT-PCR

Each sample consisted of five embryos that were flash frozen in liquid nitrogen and stored in −80 °C. Following homogenization in 350 μL TRI Reagent (Ambion), RNA was extracted with 4-bromoanisole and purified using the RNeasy Mini Kit (Qiagen). cDNA was synthesized using iScript Reverse Transcription Supermix for RT-qPCR (BioRad). qRT-PCR was performed using the TaqMan Gene Expression Master Mix (Life Technologies) on a StepOne Real-Time PCR System (Applied Biosciences). Cycling conditions were: 95 °C for 10 min; 40 cycles of 95 °C for 15 s and 60 °C for 1 min. The following primers and probes used:

ICD (5′- TCAAGTCCTCCCACGAGTATG −3′) and (5′- CTGGATCTACGTAATACGACTCAC −3′) with probe (CAGCCTCAGCGC ACCCACAT);
DBM (5′- TGCAGCAGTAGTGTCCTA −3′) and (5′- CTGGATCTACGTAATACGACTCAC −3′) with probe (CATGGAAGGACATGTATAA CGGACATTTG);
*tubb2b* (5′- GGCAGATTTTCAGACCAGACAACTT −3′) and (5′- GACCTTTGGCCCAGTTATTGC −3′) with probe (CCACTTTGACCAA ACACA);
*drosha*, (5′-ACCCCGATCGCCTTCATG −3′) and (5′-GGCTTTCAAAC TGCACTTACAGAGA −3′) with probe (ACCATCGTTCATCTGCC).

Three biological replicates (with embryos for each replicate obtained from a separate mating) were each run for each experiment. Additionally, three technical replicates were run for each RT-PCR sample. Data were normalized to drosha RNA (ΔΔCT analysis). ICD or DBM conditions were compared to the GFP control and statistical significance was determined using a two-tailed t-test in GraphPad Prism.

## 3. Results

### 3.1. Following perturbation of the Notch signaling pathway, neuronal differentiation (tubb2b expression) is initially perturbed but then recovers

**Fig. 1.**
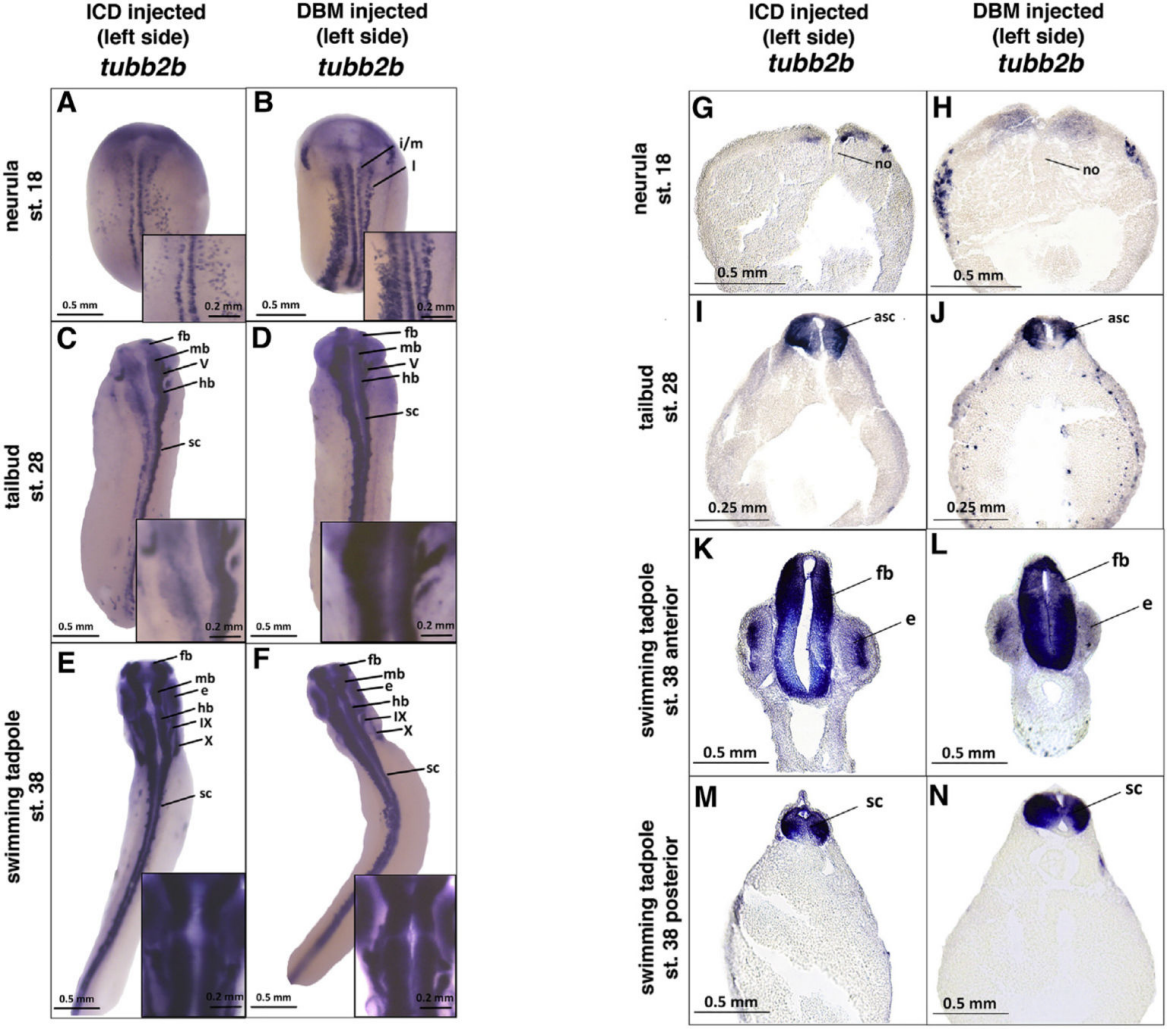
*tubb2b* expression in response to Notch perturbation. Embryos unilaterally injected with 1.5 ng ICD or DBM show perturbed expression of *tubb2b* at neurula stages on the injected (left) side (A, B, G, H). The difference in expression between sides is less stark at the tailbud stage (C, D, I, J), and minimal difference between sides is apparent at the swimming tadpole stage (E, F, K–N). All embryos shown are left-side injected. Histological analysis of *tubb2b* expression in Notch perturbed embryos supports whole-mount findings (G-N). Abbreviations: l, lateral longitudinal stripe of primary neurogenesis; i/m, intermediate/medial longitudinal stripes of primary neurogenesis; asc, anterior spinal cord; e, eye; fb, forebrain; hb, hindbrain; mb, midbrain; sc, spinal cord; V, cranial nerve V; IX, cranial nerve IX; X, cranial nerve X. For each stage and each condition, N = >100 for whole mount embryos and >15 for histological analyses.

In order to determine whether perturbing the Notch signaling pathway leads to a compensatory response, embryos were injected unilaterally at the two cell stage with 1.5 ng of the Notch IntraCellular Domain (ICD) or a DNA Binding Mutant of Suppressor of Hairless (DBM) RNA to hyperactivate or inhibit the pathway, respectively. The uninjected side served as an internal negative control. Embryos were assayed for expression of *Xenopus* neural beta tubulin (*tubb2b*), a pan-neural marker gene expressed in differentiated neurons (Oschwald et al., 1991) to assess whether embryos compensated, given that *tubb2b* gene expression is highly perturbed at neurula stages as consistently reported in the literature (Coffman et al., 1993; Ma et al., 1996; Wettstein et al., 1997). At the neurula stage (stage 18), the relative amount of differentiated neurons detected by *tubb2b* expression was noticeably lower on the ICD-injected side in accordance with previously published results (Fig. 1A, G; Chitnis et al., 1995). DBM-injected embryos show increased amounts and expanded *tubb2b* expression on the injected side at the neurula stage (Fig. 1B, H), also in agreement with previous work (Wettstein et al., 1997).

Following the effects of these perturbations over time, *tubb2b* expression appears to recover as development progresses. Tailbud stage embryos (stage 28) show an intermediate phenotype, with *tubb2b* expression appearing less perturbed than at the neural fold stage, but still noticeably diminished on the ICD-injected side (Fig. 1C, I), and increased on the DBM-injected side of embryos (Fig. 1D, J). The differences between injected and uninjected sides diminish as embryos develop to the swimming tadpole stage in both ICD- and DBM-injected embryos (Fig. 1E, F, K–N). In addition to examining *tubb2b* as a genetic marker, we also examined embryo morphology. Unlike *tubb2b* expression which was remarkably consistent within embryos from a given treatment and stage, embryos showed more variability and less dramatic compensation at the level of morphology and swimming behavior. For ICD, 84% of ICD (n = 98) embryos at tailbud stages and 82% of the embryos at swimming tadpole stages (n = 87) were in the normal or nearly normal categories. At both stages there were statistically significant differences between the ICD embryos and GFP control embryos (p < 0.05), indicating morphological compensation is not complete. For DBM, 100% of embryos at tailbud stages (n = 48) and 98% of the embryos at swimming tadpole stages (n = 50) were in the normal or nearly normal categories. Morphologically there was no difference between DBM and GFP injected controls. There were no statistically significant differences over time between tailbud and swimming tadpole stages for either ICD or DBM, suggesting that morphological compensation, to the extent that it occurs, is complete by late tailbud stages.

**Fig. 2.**
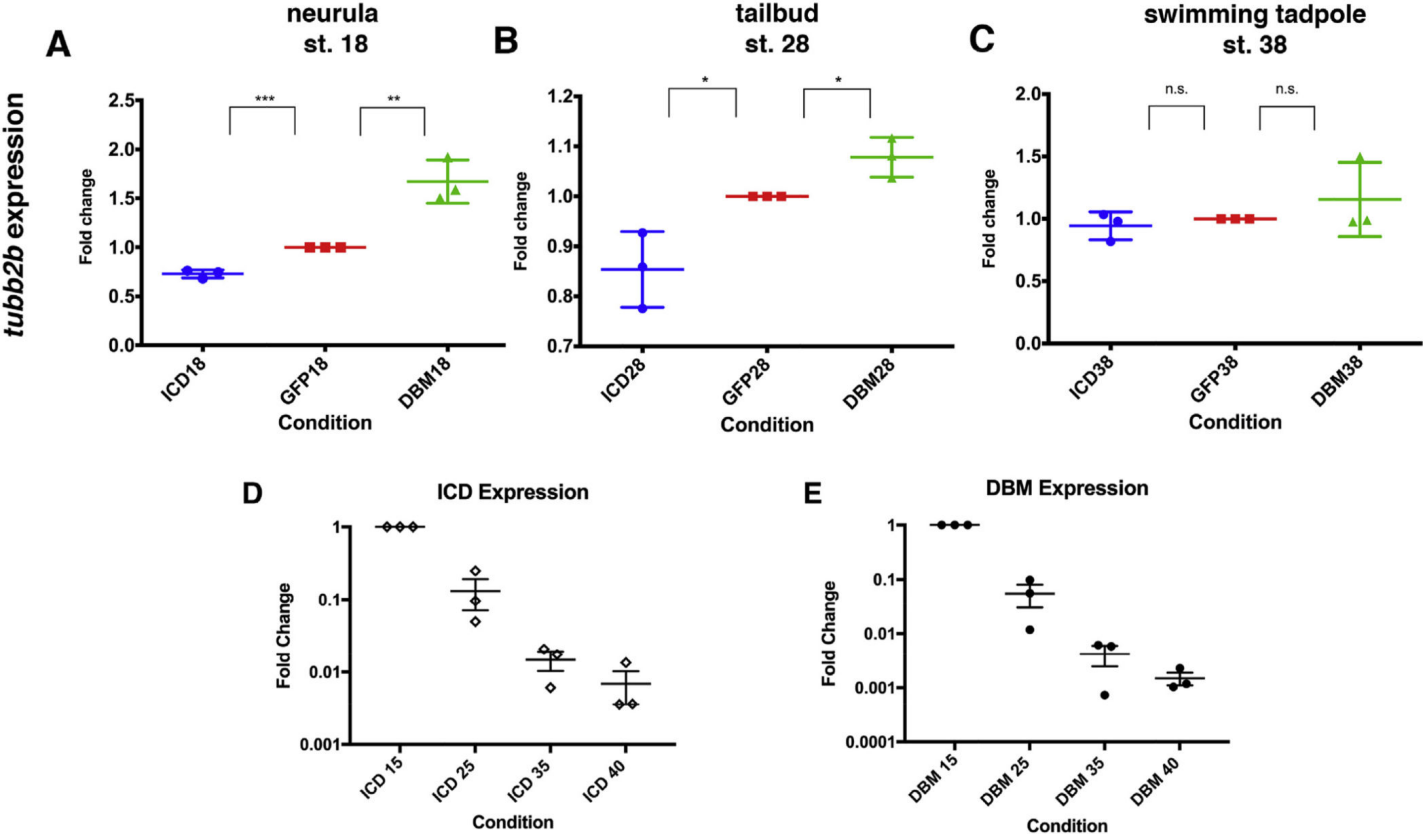
Quantification of *tubb2b* expression and construct persistence. qRT-PCR confirms that ICD injected embryos express less *tubb2b*, while DBM injected embryos express more *tubb2b* at the neurula stage (A). *tubb2b* expression remains perturbed at the tailbud stage (B), but at the swimming tadpole stage, no difference in expression level is detected when comparing perturbed embryos to vehicle-injected controls (n = 3) (C). qRT-PCR shows that while ICD (D) (n = 3) and DBM (E) (n = 3) mRNAs degrade over time, both are still detectable throughout the swimming tadpole stages. Bars represent mean ± SEM. * = p < 0.05, ** = p < 0.01.

To investigate the apparent recovery of *tubb2b* expression quantitatively, expression was assayed at three developmental stages using qRT-PCR. Consistent with the *in situ* hybridization results, at stage 18, *tubb2b* expression is significantly diminished in ICD-injected embryos while expression is significantly increased in DBM-injected embryos, both with respect to GFP-injected controls (Fig. 2A). Similar results are seen at the tailbud stage (stage 28), however the difference in *tubb2b* expression between Notch signaling-perturbed and control embryos is diminished (Fig. 2B). Finally, at swimming tadpole stages (stage 38), there is no statistically significant difference in *tubb2b* expression levels between ICD-injected embryos, DBM-injected embryos, and GFP-injected control embryos (Fig. 2C). These results quantitatively support the *in situ* hybridization results showing that terminal neuronal differentiation is initially perturbed by ICD or DBM injection, but embryos compensate over time.

To assess the amount of ICD or DBM RNA present in the embryo, qRT-PCR was performed with construct-specific primers and probes. While our experiment was designed to test the effects of a relatively acute perturbation of the Notch signaling system, with diminishing amounts of remaining construct as development proceeds, both DBM and ICD RNA are detectable even at stage 40 (Fig. 2D and E). Although significantly reduced, particularly in light of increased cell number, these results show that the embryos not only recover from the initial bolus rather than amplifying the initial effect, but can do so even in the presence of small amounts of the injected construct.

### 3.2. Bilateral perturbations and higher doses demonstrate limits to compensatory abilities

Before further investigating the nature of this compensatory response, we wished to ensure that we were using a sufficiently high dose to test the limits of the compensatory response. Although we employed a dose at the high end of those cited in the published literature (which are typically ≤ 1 ng of ICD or DBM RNA; Chitnis et al., 1995; Coffman et al., 1993; Deblandre et al., 1999, 2001; Ma et al., 1996; Moore et al., 2018; Wettstein et al., 1997; Yan et al., 2009a), we nevertheless tested unilateral injections of the two experimental constructs at a dose of 3.0 ng of ICD and DBM which resulted in poor survival and a lack of compensatory response (data not shown).

**Fig. 3.**
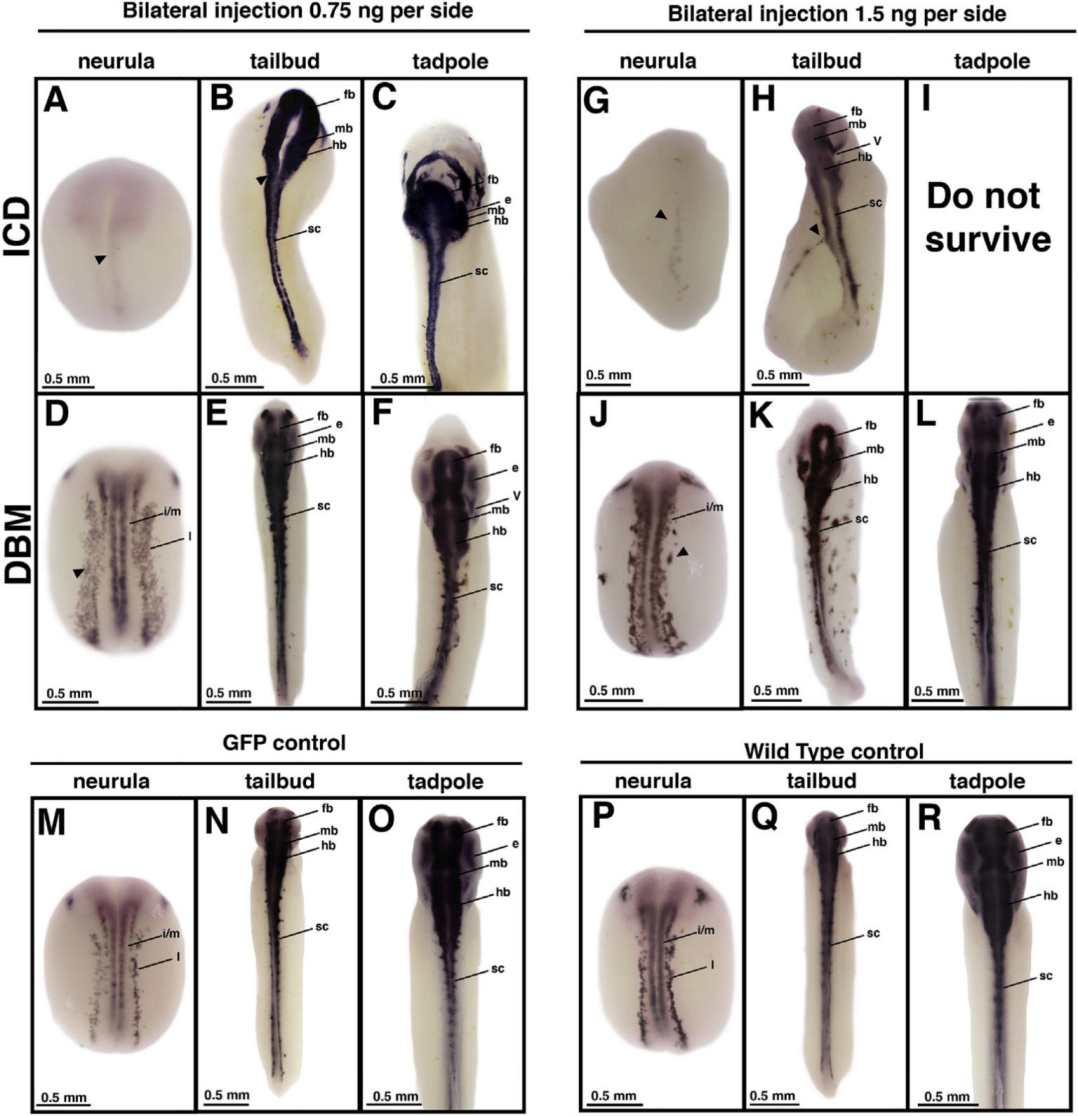
*tubb2b* expression in bilaterally perturbed embryos. Embryos were bilaterally injected at the two-cell stage with either ICD (A-C, G-I) or DBM (D-F, J-L) mRNA and reared to desired stages. ICD bilaterally injected embryos demonstrate minimal *tubb2b* expression at the neurula stage (A,G, arrowhead) and show morphological defects by the tailbud stage (B,H, arrowhead). DBM bilaterally injected embryos show ectopic *tubb2b* expression at the neurula stage (D,J, arrowhead), but appear to compensate in a gradual manner over time (E-F, K-L). GFP bilaterally injected embryos and wild type remain unperturbed (M-R). Abbreviations: i/m, intermediate/medial longitudinal stripes of primary neurogenesis; fb, forebrain; mb, midbrain; hb, hindbrain; e, eye; sc, spinal cord. For each stage, N = >50 for ICD and GFP, >10 for DBM. Embryos were obtained from a minimum of three different clutches.

Although it was important for purposes of data analysis to use the uninjected side of the embryo serve as an internal control for potential embryo or experimental variability, we also performed bilateral injections of each construct to determine if embryos would show a similar compensatory response when injected on both sides. For ICD, embryos injected with 1.5 ng on both sides showed poor survival and morphology with only 3% of the embryos displaying normal or near normal morphology at both tailbud and swimming tadpole stages, a statistically significant difference between unilateral and bilateral embryos (n = 248, p < 0.001) and between ICD embryos and GFP or wild type controls (n = >100 for all controls, p < 0.001, Fig. 3G–I, M–R). Even when 0.75 ng was injected on each side (the same total amount of RNA as for the unilateral injection), survival remained poor with only 9% of the embryos displaying normal or near normal morphology at both tailbud and swimming tadpole stages, a statistically significant difference between unilateral and bilateral embryos (n = 266, p < 0.001) and GFP or wild-type controls (n = >100 for all controls, p < 0.001, Fig. 3A–C, M–R). ICD bilaterally injected embryos show very little *tubb2b* expression at the neurula stage (Fig. 3A,G) when compared to GFP bilaterally-injected or uninjected wild type control embryos (Fig. 3M, P). They show severe neural tube defects by the tailbud stage (Fig. 3B, H) which are not observed in GFP bilaterally-injected or uninjected wild type control embryos (Fig. 3M–R). Few embryos ever survived to the swimming tadpole stage, showing that embryos bilaterally injected with ICD are not able to compensate in the same manner as ICD unilaterally injected embryos (Fig. 3C,I), suggesting a clear limit to the compensatory abilities of embryos following Notch hyperactivation. Interestingly, DBM bilaterally injected embryos (n = 20) demonstrate a compensatory response similar to that observed in unilaterally injected embryos. At the neurula stage, DBM bilaterally injected embryos expectedly show ectopic *tubb2b* expression and an expansion of the intermediate/medial longitudinal stripes of primary neurogenesis (Fig. 3D,J). Expanded *tubb2b* expression is also observed at the tailbud stage (Fig. 3E, K), but by the swimming tadpole stage, *tubb2b* expression appears nearly normal (Fig. 3F,L) when compared to GFP bilaterally injected or uninjected wild type control embryos (Fig. 3O, R).

These data suggest a limit to the embryo’s ability to mount an effective compensatory response, although there are some clear differences between ICD- and DBM-injected embryos. For subsequent analysis of the compensatory response we used the unilateral injection regimen and maximum dose (1.5 ng) reported in the published literature, a dose for which we observed consistent compensation as assayed by *tubb2b* expression.

### 3.3. Initial increase in neuronal cell number following Notch hyperactivation diminishes by swimming tadpole stages

**Fig. 4.**
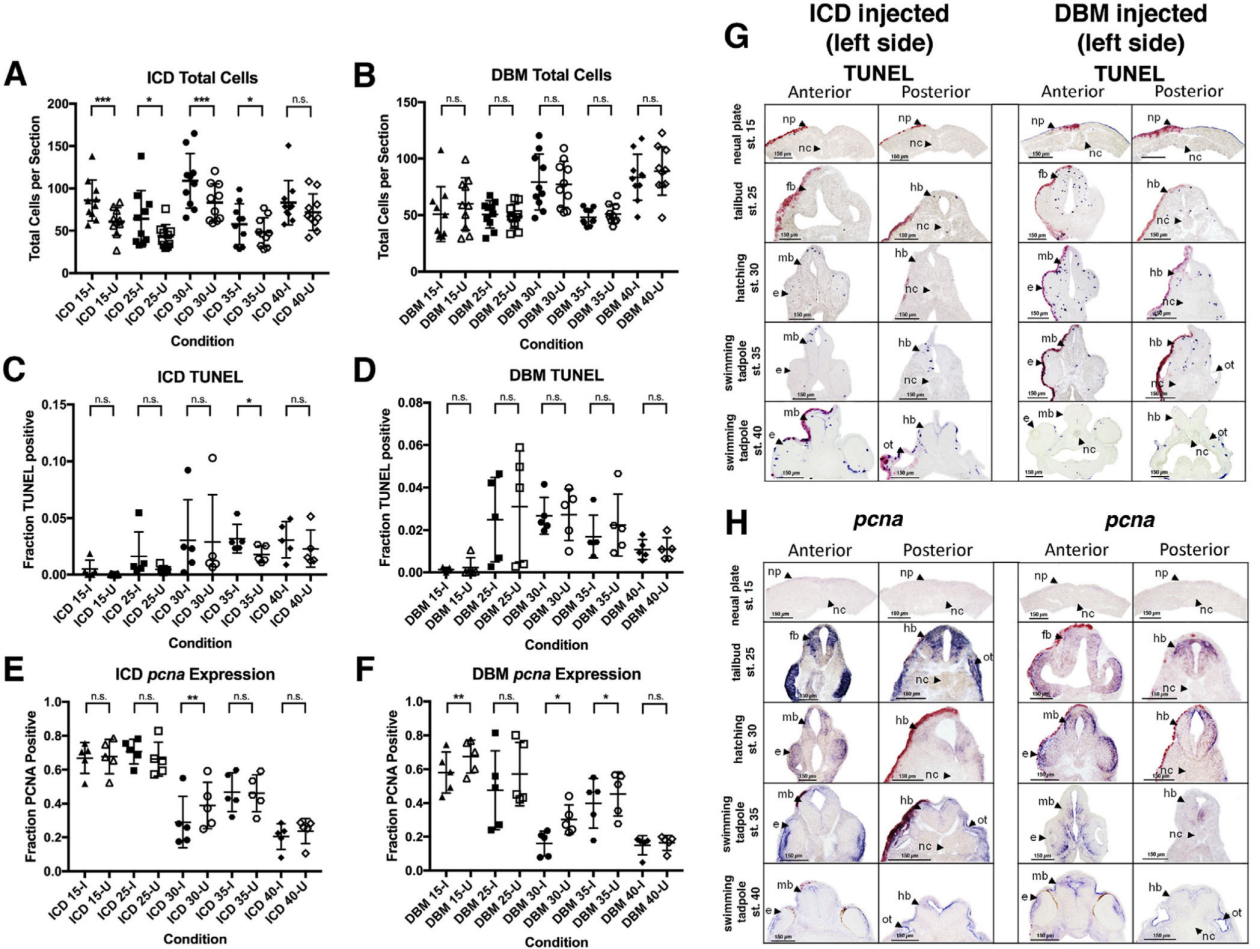
Apoptosis and proliferation in response to Notch perturbation. Total number of neural cells were measured in injected and uninjected sides of embryos over time in response to ICD injection (A) and DBM injection (B) (n = 10; * = p ≤ 0.05). Subsequently, the proportion of cells positive for TUNEL staining was measured on the injected (I) and uninjected (U) sides (C, D) (n = 5; * = p ≤ 0.05, ** = p ≤ 0.01 *** = p ≤ 0.001). Next, the proportion of neural cell expressing *pcna* was measured in injected and uninjected sides of embryos (E, F) (n = 5; * = p ≤ 0.05). Representative images of TUNEL staining and pcna expression in Notch perturbed embryos are shown in G and H, respectively. All embryos shown are left-side injected. Abbreviations: np, neural plate; nc, notochord; fb, forebrain; hb, hindbrain; mb, midbrain; e, eye; ot, otic vesicle.

In order to characterize the nature of the compensatory response we asked whether there were significant changes in cell number in the neural plate/tube by quantifying the number of cells in the anatomically defined neural region in perturbed and unperturbed sides of experimental embryos. Embryos injected with DBM do not show statistically significant differences in the total number of neuronal cells between the injected and uninjected side at any stage measured (Fig. 4B). However, embryos injected with ICD have significantly more neuronal cells on the perturbed side compared to the internal control side at neurula through early swimming tadpole stages (stages 15, 25, 30, 35) (Fig. 4A). ICD-injected embryos show no significant difference between perturbed and unperturbed sides at later swimming tadpole stages (stage 40).

### 3.4. Effects of Notch perturbation on apoptosis

In order to assess whether apoptosis contributed to the compensatory changes in neuronal cell number we analyzed the proportion of apoptotic cells on the injected and uninjected sides of Notch-perturbed embryos using a TUNEL assay (Hensey and Gautier, 1998), (Fig. 4). Embryos injected with ICD have significantly higher proportions of TUNEL positive cells on the injected side when compared to the uninjected side at stage 35 (Fig. 4C) suggesting a compensatory response to equalize cell numbers. Expectedly, DBM injected embryos displayed no significant differences in the proportion of apoptotic cells when comparing the injected and uninjected side (Fig. 4D). Representative histological sections are shown in Fig. 4G.

### 3.5. Effects of Notch perturbation on cell proliferation

Given the changes in cell number observed for ICD injections and the known interaction between Notch signaling and cell proliferation, we also assayed whether differential cell proliferation dynamics could account for the equalizing neuronal cell number between the injected and uninjected sides. To assess both the effects of Notch perturbation on cell proliferation and its role in the compensatory response, the proportion of proliferative cells to total cells was quantified in the injected and uninjected sides of Notch perturbed embryos using *pcna*, a transcript expressed in proliferating cells that encodes a DNA clamp required for DNA replication (Wullimann et al., 2005) (Fig. 4). Embryos unilaterally injected with ICD show a significantly lower proportion of *pcna* positive cells on the injected side when compared to the uninjected side at stage 30 (Fig. 4E) suggesting a compensatory response to equalize cell number. However surprisingly, embryos injected with DBM also have a significantly lower proportion of *pcna* positive cells on the injected side when compared to the uninjected side at stages 15, 30, and 35 (Fig. 4F) though this does not result in changes of cell numbers. Representative histological sections are shown in Fig. 4H.

## 4. Discussion

Robustness, the ability of organisms to respond to variations in genotypic and environmental backgrounds yet maintain similar phenotypic outcomes, is of fundamental importance for ensuring proper development given the wide range of perturbations embryos continually experience. While the particular ability of embryos to compensate for a wide range of perturbations has long been appreciated, the diversity, limitations, and mechanisms governing robustness have remained unclear (El-Brolosy and Stainier, 2017). Recently, however, there has been a renewed interest in the phenomenon of robustness in order to explain a growing number of instances in which a gene knockdown shows a severe phenotype while a mutation in the same gene shows a milder phenotype, suggesting genetic compensation in the latter case (Housden et al., 2016; Rossi et al., 2015). Recent work has shown that genetic compensation occurs in mutations generating premature termination codons by triggering nonsense-induced mRNA degradation and subsequent transcriptional compensation (El-Brolosy et al., 2019; Ma et al., 2019). Compensatory responses for mutations in other genes have shown different mechanisms of compensation, including the activation of a gene regulatory network based on a homologous/paralogous gene, and compensatory growth (Dooley et al., 2019; Ma et al., 2019).

These recent studies have largely focused on germline mutations, however there are a range of other types of perturbations that may contribute to our understanding of compensatory responses including mRNA constructs that perturb gene regulatory networks through a variety/range of mechanisms including overexpression and inhibition. Unlike germline mutations or continual exposure to a toxin, these perturbations are more transient in nature, due to ongoing degradation of the construct and dilution of injected mRNA due to cell division. However, this type of perturbation is of considerable interest for analysis of robustness. As in the case of Notch signaling perturbation, there is a dramatic alteration of both gene expression and neural plate morphology, and a subsequent compensatory response that allows the embryo to recover, although it should be noted that morphological compensation is not complete as we have shown through this work.

Here we have demonstrated a compensatory response over time to transient perturbations of Notch signaling in *X. laevis* embryos using two different injected RNA constructs, ICD and DBM, which, respectively, overexpress and inhibit Notch signaling. Many previous studies have perturbed Notch signaling to determine its role in development and our results are fully consistent with those of other studies at the early stages of development (Chitnis et al., 1995; Coffman et al., 1993; Ma et al., 1996; Wettstein et al., 1997; Deblandre et al., 1999, 2001; Lamar et al., 2001; Moore et al., 2018; Riddiford and Schlosser, 2017; Yan et al., 2009a). At neurula and tailbud stages, we observed decreased expression of *tubb2b* in embryos with hyper-activated Notch, and increased ectopic expression of *tubb2b* in embryos with Notch signaling inhibited. However, relatively few studies have followed the embryos throughout development, leaving the question of whether there is an eventual compensatory response unanswered.

We addressed this by assessing the response of embryos to Notch perturbation through the swimming tadpole stage. Others have shown genetic perturbations may change in their effect over time (Riddiford and Schlosser, 2017; Schlosser et al., 2008; Yan et al., 2009b), while some studies have reported that perturbations can persist until the swimming tadpole stages (Ochi et al., 2017; Vandenberg et al., 2012) or even through froglet stages (Jevtić et al., 2019) following RNA injection at early stages of development. Our results demonstrate a compensatory response by the swimming tadpole stage in both up-regulation and down-regulation of Notch scenarios. This appears to be an ongoing response over time given that an intermediate genetic phenotype is observed in the tailbud stage that is less severe than at the neurula stage, but not as fully compensated as in the swimming tadpole stage.

Additionally, we set out to characterize the nature of the compensatory response to identify potential mechanisms. Notch signaling is known to regulate cell proliferation (Go et al., 1998; Roese-Koerner et al., 2017), leading us to investigate neural cell numbers and proliferative status throughout the compensation. While we observed initially increased numbers of neuronal cells following Notch hyperactivation, it was normalized by the swimming tadpole stage. However, no changes in neuronal cell number were observed following Notch inhibition. This could be because active Notch signaling is associated with active proliferation (Grotek et al., 2013; Roese-Koerner et al., 2017). Accordingly, we also observed increased levels of proliferation in Notch hyperactivated embryos. After the cessation of increased proliferation, Notch hyperactivated embryos show increased levels of apoptosis, suggesting that this may be the mechanism utilized to normalize cell number during the compensation. This could be similar to the endogenous requirement for apoptosis during neural development (Offner et al., 2005; Juraver-Geslin and Durand, 2015). Together, these data suggest that the perturbation initially causes variations in cell number, caused by differential proliferation, which is then normalized by apoptosis as the compensation proceeds. However, it should be noted that while much of the literature on the Notch signaling pathway in early vertebrate development focuses on the nervous system, components of the pathway are expressed in other embryonic tissues (Peyrot et al., 2011; Castro-Colabianchi et al., 2018). Manipulations of the Notch pathway through constructs injected at early cleavage stages may be affecting as well. Thus the phenotypes we observe on neural tissue may be attributable, in part, to indirect effects exerted from the mesoderm, endoderm, and/or ectoderm. While we controlled for the uniform distribution of the tracer, it remains possible that ICD or DBM RNA was not uniformly localized and may account for some of the observed phenotypic variability.

We also tested the limits of the compensatory response and demonstrated that embryos are unable to recover from higher doses of Notch hyperactivation, likely due to toxicity. Embryos with global Notch hyperactivation, in which the ICD construct is injected into both sides of the two-cell stage embryo, did not show a compensatory response, suggesting that degradation of injected RNA alone is not sufficient to explain the observed compensation. This result also suggests that communication between both sides of the neural plate may be playing a significant role in the compensatory response. The possible interaction between the two sides of the embryo suggests that the use of the uninjected side as an internal negative control should be used with caution and comparison with wild type uninjected embryos should be considered as well.

As a whole, the data presented here reveal that embryos compensate for Notch signaling perturbation over time and can utilize mechanisms of proliferation and apoptosis to recover from unilateral Notch perturbation. While microarray analyses suggested that multiple components of the network of neural patterning genes are being differentially expressed (Vasiliu et al., 2015), further transcriptomic analyses will reveal whether nonsense-induced transcriptional compensation (El-Brolosy et al., 2019; Ma et al., 2019) and the activation of gene networks based on paralogous genes are also playing a role. This work provides another model for addressing mechanisms of genetic compensation, which could be elucidated with additional mechanistic studies based on our system.
